# Getting an Active Start: Assessing the Impact of a Physical Literacy-Based Intervention on Preschool-Aged Children’s Fundamental Movement Skills, Motor Competency and Behavioral Self-Regulation

**DOI:** 10.3390/ijerph22121861

**Published:** 2025-12-13

**Authors:** Breanne C. Wilhite, Kenneth Chui, Jennifer M. Sacheck, Daniel P. Hatfield, Margaret Morris, Megan Ziembowicz, Stephanie Herrick, Erin Hennessy

**Affiliations:** 1 Division of Nutrition Interventions, Communications and Behavior Change, Tufts University Friedman School of Nutrition Science and Policy, Boston, MA 02111, USA; breanne.wilhite@tufts.edu (B.C.W.); dhatfield@fhi360.org (D.P.H.); 2 Department of Public Health and Community Medicine, Tufts University School of Medicine, Boston, MA 02111, USA; kenneth.chui@tufts.edu; 3 Department of Social and Behavioral Sciences, Brown University School of Public Health, Providence, RI 02903, USA; jennifer_sacheck@brown.edu; 4 FHI 360, Washington, DC 20037, USA; 5 Tufts University School of Occupational Therapy, Medford, MA 02155, USA; margaret.morris@tufts.edu (M.M.); megan.ziembowicz@tufts.edu (M.Z.); 6 Rising New York Road Runners, New York Road Runners, New York City, NY 10019, USA; sherrick@nyrr.org

**Keywords:** physical literacy, self-regulation, fundamental movement skills, gross motor competence, early childhood development, childcare

## Abstract

Fundamental movement skills (FMS) and behavioral self-regulation (SR) are important for lifelong physical activity (PA). While physical literacy (PL) mediates child PA, its broader developmental impact in early childhood education (ECE) remains underexplored. The Active Start feasibility study examined a 10-week PL-based intervention’s effects on FMS (stationary, locomotion, object control), total motor competency and behavioral SR, as well as sex-based differences, among 3–5-year-olds in Somerville, Massachusetts childcare centers. Children (mean age = 3.8 years, 55% boys) were randomized by childcare center (two per condition) into intervention (*n* = 39) or control (*n* = 35) groups. Outcomes were measured at baseline and final using the Peabody Developmental Motor Scales for FMS and motor competency and the Head–Toes–Knees–Shoulders task for SR. Intervention effects were assessed using linear mixed-effects and zero-inflated mixed-effects hurdle models, with interactions examining sex-based differences in program effectiveness. Stationary skills had a net average improvement of 2.3 points in the intervention group compared to the control (*p* < 0.01). No significant treatment effects were observed for locomotor, object control, total motor competency or behavioral SR skills (*p* > 0.05). The treatment effects did not significantly differ by sex. PL-based ECE interventions may enhance stability skills in motor development, but further research in larger samples is needed to determine broader impacts on early childhood development.

## 1. Introduction

Early childhood is a critical period for developing skills essential for lifelong physical activity (PA) and health [1,2]. Fundamental movement skills (FMS), the “building blocks” of advanced movements, include locomotor (e.g., running, hopping), object control (e.g., catching, kicking) and stability (e.g., balance, body control) skills [3,4]. Gross motor competence, or FMS proficiency, supports physical, cognitive and social development and promotes PA across the lifespan [5,6,7]. Self-regulation (SR) skills, which begin to develop between ages 3 and 5, are key to adaptive development and school readiness [8,9,10]. Behavioral SR, encompassing executive functioning components like attention, cognitive flexibility, working memory and inhibition, plays an important role in acquiring health behaviors, including PA [11].

Gross motor competency in early childhood develops through structured instruction, feedback and quality experiences [12,13,14,15]. Similarly, SR is actively shaped by environmental interactions rather than maturing innately over time [16,17,18]. Research shows that early childcare and education (ECE) interventions can enhance both gross motor competency [19,20,21] and SR [22,23] in preschoolers. Despite their importance for healthy development and lifelong PA, these skills are often studied separately. More research is needed on if and how ECE interventions impact both motor and cognitive skills [24,25]. Few studies have examined the combined effects of ECE interventions on motor and cognitive skills, highlighting a gap in the literature [8,26].

Physical literacy (PL) mediates child PA by integrating three domains of development: cognitive (PA knowledge), social-emotional (motivation, self-efficacy, confidence) and motor (FMS) [27]. A physically literate child, equipped with movement competency and diverse activity proficiency, is more likely to lead an active lifestyle than their non-physically literate peers [28]. PL is increasingly studied for its role in promoting early childhood PA [22,23,29,30,31,32], yet its holistic impact on development and practical application in ECE interventions remains underexplored.

In general, girls tend to have lower average motor competency than boys [33], while boys tend to have lower SR [34,35]. Sex disparities in gross motor competency and SR among preschoolers may contribute to PA differences (i.e., boys engage in higher levels of PA than girls at all ages) [29,33,36,37,38,39,40], yet little research explores strategies to reduce these gaps in early childhood environments [41,42]. Evidence suggests that boys and girls may respond differently to PA interventions [43,44,45], highlighting the need to study sex-specific responses in ECE interventions as a first step toward addressing these disparities.

This study aimed to address gaps in ECE intervention research by evaluating a PL-based intervention (comprising twice weekly PL lessons, active free play and literacy activities) on preschoolers’ FMS, total motor competency and behavioral SR. A secondary aim was to assess whether treatment effects differed by sex. This 10-week feasibility study was conducted in four childcare centers in Somerville, Massachusetts. We hypothesized that children in intervention childcare centers would have greater increases in gross motor skills and SR test scores than those in control centers. Additionally, we expected greater improvements in gross motor skills for girls and SR for boys compared to their counterparts.

## 2. Materials and Methods

In spring 2018, directors of 12 licensed childcare centers in Somerville, MA, were contacted to assess interest and seek permission to recruit childcare providers at each center. Study staff reached out to providers via phone, email and in-person presentations covering the intervention, provider training, study evaluation days, compensation and parental consent. Six centers showed interest, and four participated—two were randomized to receive the intervention and two were randomized to the control condition (delayed intervention). Centers were stratified by income, using childcare tuition as a proxy, prior to randomization. Data collection occurred at all centers from June to September 2018.

Families were recruited via study flyers sent home in backpacks, parent nights with presentations by research staff and in-person interactions during pick-up and drop-off times, with materials available in English, Spanish and Portuguese. Of 151 eligible children (ages 3–5), 88 parents provided consent. Parents provided consent for their child in person or returned consent forms to centers, which were then securely transferred to research staff. While all children at intervention sites received the intervention, only those with parental consent participated in data collection.

### 2.1. Physical Literacy Program Development

The intervention included 20 lessons, aiming for 2 per week over 10 weeks. Each 30 min lesson featured an FMS with related activities. Families received hard-copy newsletters with lesson details and at-home activities. In addition to the lesson, providers facilitated a 15 min active free play session and a 15 min dialogic literacy activity, totaling 60 min of structured activities per day. These could be completed together or spread throughout the day. Families also received all 10 books used in literacy activities. The total intervention dose was approximately 1200 min (60 min/day × 2/week × 10 weeks).

Grounded in ECE intervention research, the program was co-created by child PA and development experts and was designed to address all three domains of PL concurrently. Lessons were adapted from Rising New York Road Runners’ curriculum (grades pre–K). The adapted curriculum was aligned with national PE standards, specifically highlighting constructs of PL [46]. The curriculum integrated movement concepts for motor and cognitive development [32,47] and included social–emotional components through structured instruction and feedback [12,13,14,15]. Active free play components targeted motor and social–emotional growth [48,49,50], while interactive storybooks supported cognitive and motor development [51,52]. The intervention aimed to lay the foundation for healthy development and lifelong PA engagement (see Table 1).

### 2.2. Provider Training and Implementation Procedures

Research staff trained intervention-group providers before child recruitment through a three-hour session focused on improving providers’ knowledge of PL and motor skill development and improving providers’ self-efficacy for intervention delivery. Research staff provided modeled lesson plans and individual support to help providers integrate intervention activities into classroom routines.

During implementation, the Primary Investigator (PI) and co-PI led lessons for five weeks while providers shadowed them, then providers took over with research staff observing for the remaining five weeks. Research staff provided up to 1 h/week of assistance to ensure fidelity of intervention delivery; quality was observed but not systematically recorded. Intervention-group providers committed up to 5 h for training and 4 h/week for lesson prep and delivery during the intervention. All providers received a USD 40 gift card, and participating centers received PA equipment (e.g., new hoops, balls, bean bags, etc.), two copies of each book and a USD 100 gift card.

### 2.3. Measures and Instruments

Parents reported child’s sex (male or female), birth date and race/ethnicity. Children underwent two 60 min measurement visits by trained research staff, assessing height and weight (baseline only), motor competency and behavioral SR. Baseline assessments occurred before implementation (May–June 2018), with final assessments post-intervention (August–September 2018). Research staff were trained by experts in anthropometry, motor competency and SR assessment. Tufts University occupational therapy graduate students conducted motor competency assessments after training, which included didactic instruction, applied practice and field measurements to establish inter-rater reliability.

Children’s height and weight were measured privately in triplicate by stadiometer measurement devices and Seca digital scales, respectively, with trained staff recording data on paper forms. BMI and BMI z-scores were calculated from baseline measurements.

Children’s gross motor competency was assessed using the Peabody Developmental Motor Scales–Second Edition (PDMS-2) [53], a standardized, validated tool for measuring interrelated abilities of early motor development in children from birth to five. The PDMS-2 includes 249 items across six subtests (reflexes, stationary, locomotion, object manipulation, grasping and visual-motor integration), with each item scored 0–2. Subtest points are summed (raw score) and converted into a standard score based on age and sex, with higher scores indicating better motor performance [54]. Standard scores of 7 to 13 are considered “average,” and the gross motor quotient (GMQ)—the sum of stationary, locomotor and object manipulation standard scores—ranges from 85 to 115 for “average motor development.” Since standardized scores are recommended for comparing skills across ages and sexes, we used standard scores in this study [36].

The Head–Toes–Knees–Shoulders (HTKS) task was used to assess behavioral SR, measuring working memory, cognitive flexibility and inhibitory control) [55]. This reliable, validated tool for diverse groups of preschoolers [56,57] involves responding to prompts by touching the opposite body part (e.g., “touch your head” requires touching toes) [58]. HTKS consists of three sections with 4–10 tests each. Children earn 2 points for correct responses, 1 for self-corrections and 0 for errors. They advance to the next section with at least 4 points. Total scores (0–60) reflect behavioral SR, with higher scores indicating stronger behavioral SR.

### 2.4. Post Hoc Detectable Effects Calculations

This analysis was exploratory in nature. Here we report the detectable effects for the outcomes, estimated using the empirical baseline standard deviations and Pearson correlation coefficients between the two timepoints. Based on group sizes of 39 and 35, we had 80% power to detect effects more extreme than 1.67 in stationary scores, 1.10 in object manipulation scores, 1.48 in locomotion scores, 5.90 in GMQ and 9.72 in SR.

### 2.5. Statistical Analyses

Statistical analyses were conducted in Stata 19 with a Type I error rate set as 0.05. Descriptive statistics (frequencies and percentages for categorical variables; means and standard deviations for continuous variables) summarized sample characteristics at baseline by sex and outcomes at baseline and final assessments by sex. Pearson chi-squared tests assessed differences in race, ethnicity and sex across treatment groups at baseline. Independent samples *t*-tests examined between-group differences in age, height, weight, BMI and BMI z-scores at baseline. Independent samples *t*-tests examined between-group differences in all outcome variables at baseline and final.

The intervention’s impact on total motor competency and FMS subtests (stationary, object manipulation, locomotion) was estimated using three linear mixed-effects models. Independent variables included treatment exposure (binary), timepoint (binary), and their interaction, while dependent variables were total GMQ and subtest standard scores. Mixed models accounted for repeated measures, with each student as a random intercept.

The first model assessed unadjusted treatment effects. Based on the literature [36,40,59,60], the second and third models adjusted for age (months), sex and BMI. As the baseline values of the dependent variables were found to predict longitudinal change, we also adjusted for baseline values. The third model added interaction terms (treatment × timepoint, treatment × sex, timepoint × sex, and treatment × timepoint × sex) to assess differential treatment effects by sex. Two sensitivity analyses were conducted for models two and three. One used complete cases to assess bias from missing data, including only children with full data for each subtest. Another reexamined models after excluding extreme high and low baseline scores, retaining only children with “average” baseline values (per the literature base [36]) to evaluate differences in treatment effects.

Initial analysis showed 7% of students scored zero on SR at baseline (*n* = 68) and 18% at final (*n* = 62). Due to non-normal distributions and zero inflation, we used five zero-inflated mixed-effects hurdle models [61,62] to assess treatment effects on behavioral SR. These models include: (1) a binary logistic component estimating the likelihood of a non-zero SR score and (2) a negative binomial component estimating SR score increases among those with non-zero scores, both accounting for repeated measures. The negative binomial model was chosen due to data under-dispersion as assessed by alpha statistics. The first and third models assessed unadjusted treatment effects, while the remaining three adjusted for sex, age (months), BMI and baseline SR scores. Student’s study ID numbers were included as random intercepts. The fifth model included a three-way interaction (treatment × timepoint × sex) to assess differential treatment effects by sex.

Predictive margins from linear mixed-effects and mixed-effects negative binomial models were used to compute adjusted means for all outcomes at each timepoint and across treatment groups, holding covariates at average. These estimates illustrate the effects of treatment over time.

## 3. Results

**Figure 1:**
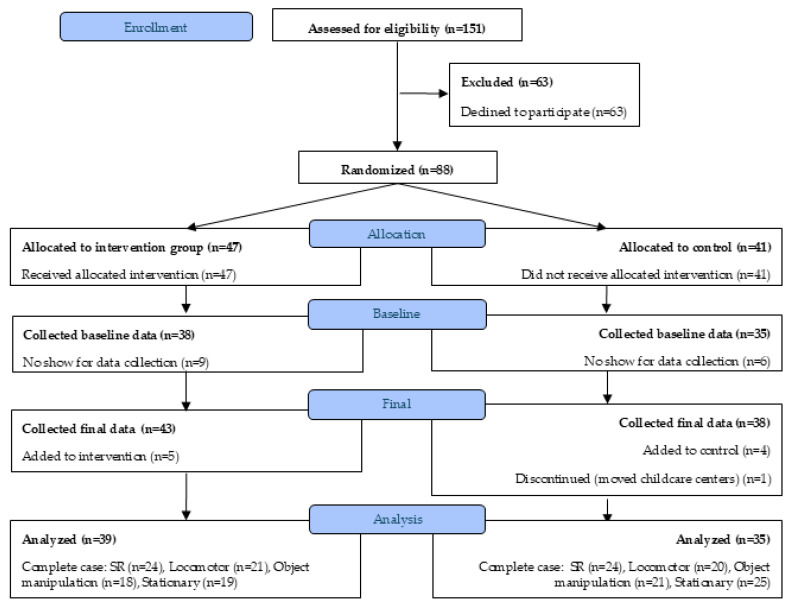
CONSORT diagram for Active Start study participation.

Four childcare centers participated, with 88 children consented (*n* = 47 intervention, *n* = 41 control). See Figure 1 for the CONSORT diagram. After excluding cases with missing demographic or anthropometric data, 74 children (*n* = 39 intervention, *n* = 35 control) remained for analysis. Complete case sample sizes varied by outcome: SR (*n* = 48), locomotor skills (*n* = 41), object manipulation skills (*n* = 39) and stationary skills (*n* = 44).

Treatment groups were similar in race, ethnicity, sex, age, height and weight (Table 2). Differences in BMI and BMI z-scores were not statistically significant (*p* = 0.09, *p* = 0.08). Boys and girls were comparable across all characteristics.

Baseline outcome measures did not differ significantly between groups, with mean scores falling in the “average” range for both (Table 3). Locomotor scores were highest, followed by stationary and object manipulation skills. At baseline, control group boys scored significantly higher in stationary skills (10 vs. 7.5, *p* = 0.01), object manipulation (9.6 vs. 8.1, *p* = 0.04) and GMQ (101.2 vs. 89.2, *p* = 0.01) than intervention boys. No significant baseline differences were found for girls, but at the final stage, intervention girls scored significantly higher in stationary skills (12 vs. 10.2, *p* = 0.03) and GMQ (106.7 vs. 94.1, *p* = 0.03) than control girls.

### 3.1. Interventions Effects on FMS and Total Motor Competency

Table S1 presents the linear mixed-effects model results. The non-significant three-way interaction (treatment × timepoint × sex) suggests the intervention effect did not differ by sex, so the adjusted Model 2 was used for interpretation. Model 2, adjusted for sex, age, BMI and baseline outcome values, showed that stationary skill scores had a net average improvement of 2.3 points in the intervention group compared to the control group (*p* < 0.01). No significant treatment effects were found for locomotor, object control or total motor competency scores (*p* > 0.05). Sensitivity analyses (complete case and exclusion of extreme values) yielded similar results, with no meaningful differences, to Model 2.

**Figure 2:**
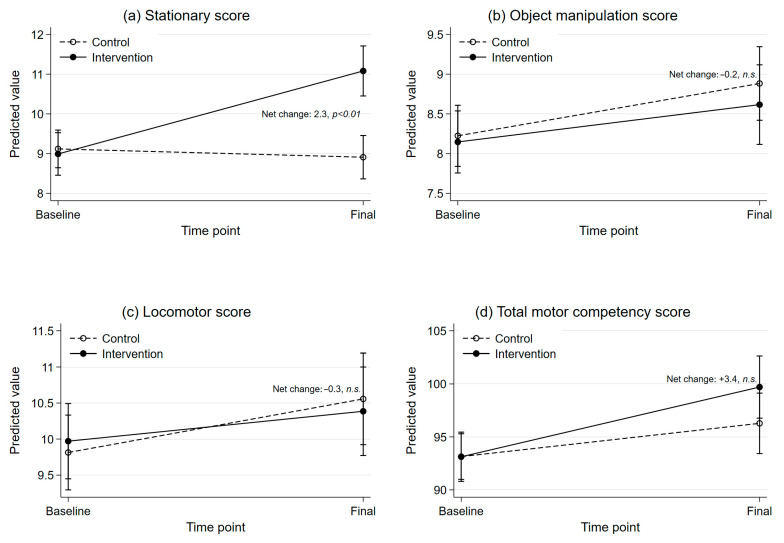
Predictive means for motor skills across time and according to treatment group, adjusted for child sex, age and BMI at baseline and baseline values. Solid lines represent the intervention group; dashed lines represent the control group. Error bars represent 95% confidence intervals: (**a**) stationary score adjusted means; (**b**) object manipulation score adjusted means; (**c**) locomotor score adjusted means; (**d**) total motor competency score adjusted means.

Figure 2 presents predictive mean scores over time by treatment group, reflecting adjusted mean outcomes based on Model 2. The slope of each line represents the group specific mean change in scores from baseline to final. The difference between the slopes of the two lines represents the net effect. For stationary skills (panel a), intervention group Δ = +2.1, control group Δ = −0.2 (net Δ = +2.3, *p* < 0.01). For object manipulation skills (panel b), intervention group Δ = +0.5, control group Δ = +0.7 (net Δ = −0.2, n.s.). For locomotor skills (panel c), intervention group Δ = +0.4, control group Δ = +0.7 (net Δ = −0.3, n.s.). For total motor competency (panel d): intervention group Δ = +6.6, control group Δ = +3.1 (net Δ = +3.4, n.s.).

### 3.2. Intervention Effects on Behavioral SR

Table S2 presents the mixed-effects hurdle model results for behavioral SR. The binary component, predicting the likelihood of a non-zero score, showed no significant difference for the intervention group at the final stage, compared with the control group. Adjusting for covariates (Model 2) did not alter findings. The positive component, assessing SR improvement among those with non-zero scores, found no significant treatment effect, and results did not differ by sex (Model 5). Therefore, Model 4 was interpreted. Overall, the intervention did not significantly impact behavioral SR at the final stage (IRR = 0.98, n.s.).

**Figure 3:**
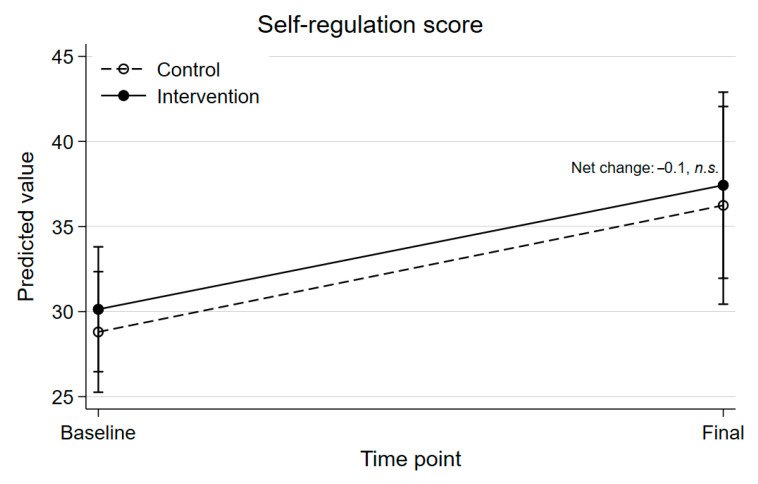
Predictive means for behavioral self-regulation, given nonzero scores, across time and according to treatment group, adjusted for child sex, age and BMI at baseline and baseline values. Solid lines represent the intervention group; dashed lines represent the control group. Error bars represent 95% confidence intervals.

Figure 3 shows the predictive mean scores across time according to treatment group for behavioral SR from Model 4. For SR skills, intervention group Δ = +7.3, control group Δ = +7.4 (net Δ = −0.1, n.s.).

## 4. Discussion

This study examined the impact of a PL-based intervention on FMS, total motor competency and behavioral SR among preschoolers, while assessing differential treatment effects by sex. The results indicated that the intervention led to a statistically significant improvement in stationary skills, with an average net gain of 2.3 points in the intervention group compared to the control group. However, no significant intervention effects were observed for locomotor skills, object control skills, total motor competency or behavioral SR. Additionally, the treatment effects did not significantly differ by sex. Implications for PL-based ECE interventions, future directions and the limitations of the study are discussed further below.

Our findings make a novel contribution to the literature on ECE interventions focused on promoting motor development. While prior studies show that movement-based interventions can enhance FMS [24,63,64,65,66,67,68,69], most focus on total motor competency or locomotor and object control skills [1,64,70,71], often overlooking stability (stationary) skills in intervention design and measurement procedures [5]. Our study addresses this gap by explicitly measuring and reporting on stability skills in an ECE intervention.

Previous research suggests that FMS may develop at different ages or rates, with locomotor skills typically emerging earlier, around age three, and developing more rapidly [72], while object control skills tend to mature later, often beyond age five [73,74]. This developmental trajectory may partially account for our findings, as the age range of the participating preschoolers may not have aligned with the optimal developmental window for detecting significant changes in some skill areas. Further research is warranted to explore how developmental timing and influencing factors, such as sex [75], may shape skill acquisition. Such insights could inform the design and timing of ECE interventions to promote motor competence more effectively in preschoolers.

The intervention’s delivery model (five weeks by research staff, then five weeks by childcare providers with research staff observation) and the sequencing of stationary skills (lessons 1–5) may have influenced the findings. Research indicates that teacher education level affects teaching quality and motor skill development in ECE settings [76,77], though its impact on stationary skills is less studied. Given research staff’s expertise in child development and motor skills, their instruction may have contributed to the significant stationary skill improvements. Future research should examine whether treatment effects differ when childcare providers deliver all ten weeks. Additionally, children may have been more responsive early in the intervention when stationary skills were introduced, potentially influencing results and suggesting a need for further research.

The ten-week intervention duration, while appropriate for a feasibility study and consistent with other brief ECE interventions [19,20,21], may have provided insufficient time for children to develop and demonstrate measurable improvements in all motor skill domains. Research suggests that motor skill interventions of longer duration—typically ranging from 6 months to one year—tend to show more robust and comprehensive effects across multiple FMS domains [64]. Object control skills, which typically develop later than locomotor skills [73,74], may require extended practice periods to achieve detectable changes at the population level. Similarly, behavioral SR skills, which undergo rapid but variable development during the preschool years [8,9,10], may benefit from longer intervention exposure to produce measurable effects on standardized assessments like the HTKS.

Although our intervention did not significantly impact SR scores, findings align with prior research [62,78], highlighting the challenge of improving SR in ECE interventions. Prior studies have noted HTKS floor effects, possibly due to competence–performance distinctions (i.e., children possess skills but struggle to demonstrate them on demand) [79,80]. Although validated in diverse populations [57], recent modifications to HTKS aim to address these limitations [56,81], and combining HTKS with teacher evaluations may improve assessment accuracy [80,82]. Future research should explore how different and additional SR assessment methods influence findings.

Most ECE interventions targeting behavioral SR integrate multiple SR domains or focus on school readiness, limiting direct comparisons of our findings to the current literature base [80,83,84,85,86]. Movement-based ECE interventions show potential for improving SR [62,78,87,88,89,90], though the studies testing those interventions primarily measure cognitive SR [90] or executive functioning separately, complicating generalizability to behavioral SR [78,88,89]. To our knowledge, this is the first study examining a PL-based intervention’s impact on behavioral SR, contributing to a gap in the ECE literature [24,62].

While FMS score trends vary across populations [24,36,37], our baseline findings are consistent with prior research indicating that boys tend to excel in object manipulation skills [37,91,92], locomotor skill differences are mixed [60,92] and girls generally outperform in balance/stability tasks [40,60]. These patterns suggest that our sex-stratified baseline values are consistent with those observed in other studies, supporting the potential generalizability of our sex-based findings to similar populations and contexts.

Treatment effects did not significantly differ by sex across outcomes. This null finding warrants deeper consideration given that the developmental literature consistently documents sex differences in both motor competency and SR during early childhood. As noted earlier, boys typically demonstrate advantages in object manipulation skills while girls often excel in balance and stability tasks [37,40,60,91,92], and boys tend to show lower self-regulation compared to girls during preschool years [34,35]. These well-established patterns emerge from a complex interplay of biological factors (e.g., differences in muscle composition, body proportions, maturation rates), sociocultural influences (e.g., differential encouragement and opportunity structures for specific activities, gender-typed toy and activity preferences) and environmental contexts (e.g., availability of equipment and play spaces, peer interactions) that shape motor and cognitive development throughout early childhood [75,93,94].

Given these documented sex differences in the literature, the absence of differential intervention effects in our study may reflect several possibilities. First, our intervention was intentionally designed to provide balanced exposure across FMS domains rather than emphasizing skills where one sex typically excels. This design may have created learning environments where both boys and girls had equal opportunities to develop areas of relative strengths and weaknesses. The structured nature of the intervention—with explicit instruction, practice opportunities, and feedback across all skill domains—may have minimized the impact of informal socialization processes that often contribute to sex differences in naturalistic settings.

The preschool age range represents a developmental period characterized by rapid but highly variable skill acquisition, where individual differences within sex groups often exceed average differences between them [72,75]. Our small sample size and relatively brief intervention duration may have been insufficient to detect subtle sex-specific responses that might emerge with longer exposure or in larger samples. Moreover, developmental perspectives increasingly emphasize that observed sex differences in motor skills often reflect differential opportunity and socialization rather than innate capacity [40,95], and are more pronounced in unstructured, free-play contexts compared to structured learning environments with intentional instruction like ours [96].

While limited research on ECE interventions suggests they may not impact motor competency differently by sex [42], sex-specific effects have been observed for SR and executive functioning [97,98]. Among school-aged children, girls tend to favor educational interventions while boys respond better to environmental ones [43,45], with overall trends showing greater intervention benefits for girls [44]. However, sex-specific responses to movement-based interventions in preschoolers remain understudied, with one study suggesting greater improvements in PA in girls due to lower baseline PA levels [99].

Future research should examine (a) whether sex-specific intervention effects emerge with adequately powered samples and longer durations; (b) whether targeted additional practice in domains where individual children show relative weakness (regardless of sex) yields benefits while maintaining equitable outcomes; (c) how implicit caregiver and teacher beliefs about sex-based abilities may inadvertently reinforce differences in children’s motor and SR development; and (d) whether specific intervention features (e.g., activity group composition, competitive vs. cooperative activities, teacher interaction patterns) moderate sex-specific responses. From a practice perspective, our findings suggest that ECE interventions with balanced exposure, explicit instruction and equitable access may support motor and SR development across all children without widening sex-based gaps—an important equity consideration for early childhood programming.

### Study Limitations

Our measures focused primarily on motor development rather than all domains of PL due to the lack of reliable and valid instruments for assessing other domains in this young age group at the time when the study was conducted. Additionally, the pilot nature of this study constrained our ability to assess numerous outcomes simultaneously. At baseline, children in our study demonstrated slightly higher-than-average gross motor skills, with 55.4% scoring in the “average” range or above in total motor competency and 70.3% scoring “average” or above on subtests compared with the U.S. norm (50%) [36,37]. This baseline performance suggests our population may have had stronger motor abilities than typical, potentially limiting detectable intervention effects. Moreover, given that the study sample was powered to detect only moderate or larger improvements, more subtle subgroup or domain-specific effects were unlikely to be observed.

The relatively brief intervention duration (10 weeks) may have limited our capacity to detect meaningful changes in some developmental domains. While this duration was appropriate for establishing feasibility, longer interventions may be needed to impact skills that develop more gradually or require sustained practice, such as object control and behavioral SR. The concentration of stationary skill instruction during the first five weeks, when research staff led lessons, may have contributed to the observed effects in this domain but not others. Future research should examine optimal intervention duration and sequencing strategies to maximize developmental benefits across all targeted domains.

Additionally, although we collected data on family-, provider- and preschool-level factors known to influence motor competency and SR development [93], these variables were not included in our statistical models. For example, factors such as smaller child-to-classroom ratios have been shown to support gross motor skill development [77]. However, given that our intervention and control groups were drawn from preschools with comparable environments, including similar socioeconomic profiles and childcare tuition rates, the variability in these contextual factors was likely limited.

Taken together, these findings highlight both the promise and the limitations of embedding PL-based programming within ECE environments. This study also demonstrates the feasibility of training childcare providers to deliver PL-based activities within existing classroom schedules and curricula. Importantly, the program design and training were conducted in partnership with New York Road Runners, which offers free resources and support that can facilitate broader implementation. These findings suggest that PL-based interventions can be realistically embedded into ECE settings when provider training, curricular alignment and community partnerships are leveraged.

## 5. Conclusions

Designing ECE interventions to enhance motor competency and SR in preschoolers is increasingly prioritized due to the association between these skills and positive health behaviors like PA. Findings from the Active Start study show the PL-based intervention improved stationary skills but no other FMS, total motor competency or behavioral SR, with no sex-based differences in intervention effects. These results suggest that PL-based interventions may positively support the development of stability skills in young children.

Future research is needed to confirm effectiveness in larger trials, examine subgroup differences (including potential sex-specific responses) and assess longer-term outcomes. Additional work should also investigate the timing and rate of FMS development, optimize delivery models and strengthen SR assessment through multi-method approaches that combine direct observation with teacher evaluations. Collectively, such efforts will be essential for determining whether PL-based ECE interventions can be scaled and sustained to improve motor skills and SR across diverse early-childhood populations.

## Figures and Tables

**Table 1:** Active Start intervention components, supporting research and delivery details.

Component	Length	Dose *	Description	PL Domains	Details
Physical literacy (PL) lesson	30 min	*n* = 20	Featured a “skill of the week” with corresponding activities that were intended to be fun, engaging and appropriate across developmental levels. Participating families received a hard-copy newsletter describing the lessons and how to incorporate PL-based activities at home.	Motor, cognitive and social–emotional development	Lessons 1–5: stationary skills Lessons 6–12: locomotor skills Lessons 13–20: object manipulation skills
Active free play	15 min		Type of PA where children are allowed to move their bodies in a way that they choose. This is also known as unstructured PA.	Motor and social–emotional development	
Literacy activity	15 min	*n =* 10 books	Included interactive storybooks that were complementary to the program activities and presented the PL concepts in an age-appropriate format using dialogic literacy strategies. Participating families received a new book each week that was featured during the literacy activity.	Cognitive and motor development	*n* = 8 promoted PA *n* = 6 promoted gross motor skills *n* = 1 promoted self-regulation

* Each intervention component was delivered twice a week.

**Table 2:** Baseline characteristics of 74 preschool-aged children in Active Start, N (%) or mean ± SD.

	Full Sample			Boys			Girls		
	Control	Intervention	*p*-Value ^1^	Control	Intervention	*p*-Value ^1^	Control	Intervention	*p*-Value ^1^
N	35	39		18	23		17	16	
Race			0.76			0.68			0.65
Multiracial	2 (7%)	3 (8%)		1 (7%)	1 (5%)		1 (6%)	2 (13%)	
White	28 (90%)	31 (84%)		14 (93%)	21 (91%)		14 (88%)	11 (73%)	
Black	0 (0%)	1 (3%)		0 (0%)	0 (0%)		0 (0%)	1 (7%)	
Asian	1 (3%)	2 (5%)		0 (0%)	1 (5%)		1 (6%)	1 (7%)	
Ethnicity			0.55			0.50			1.00
Non-Hispanic	29 (94%)	34 (89%)		14 (93%)	19 (86%)		15 (94%)	15 (94%)	
Hispanic	2 (6%)	4 (11%)		1 (7%)	3 (14%)		1 (6%)	1 (6%)	
Sex									
Boys	17 (49%)	23 (59%)	0.42	-	-		-	-	
Girls	17 (49%)	16 (41%)		-	-		-	-	
Age (years)	3.8 ± 0.7	3.8 ± 0.8	0.98	3.8 ± 0.7	3.9 ± 0.8	0.70	3.8 ± 0.6	3.7 ± 0.8	0.59
Age (months)	51.7 ± 8.4	51.6 ± 9.5	0.91	51.0 ± 9.3	51.8 ± 10.4	0.79	52.5 ± 7.7	51.0 ± 8.5	0.60
Height (cm)	105.1 ± 5.8	103.4 ± 6.9	0.26	103.4 ± 5.0	103.9 ± 7.3	0.82	106.9 ± 6.2	102.7 ± 6.6	0.07
Weight (kg)	17.5 ± 2.3	17.6 ± 2.8	0.96	16.8 ± 1.5	18.06 ± 3.3	0.17	18.3 ± 2.8	17.0 ± 1.7	0.12
BMI	15.8 ± 1.2	16.4 ± 1.5	0.09	15.7 ± 1.1	16.5 ± 1.6	0.07	15.9 ± 1.3	16.1 ± 1.2	0.71
BMI z-score	0.2 ± 0.9	0.6 ± 0.9	0.08	0.02 ± 0.9	0.6 ± 1.0	0.07	0.4 ± 0.8	0.5 ± 0.8	0.60

^1^ Intervention and control groups were compared using Pearson chi-square tests for categorical variables (race, ethnicity and sex) and independent samples *t*-tests for continuous variables (age, height, weight, BMI and BMI z-score).

**Table 3:** Baseline and final scores by treatment group in Active Start, mean ± SD.

		Baseline			Final		
	Score ^2^	Control (*n* = 35)	Intervention (*n* = 39)	*p*-Value ^1^	Control (*n* = 40)	Intervention (*n* = 42)	*p*-Value ^1^
Full	Stationary	9.5 ± 2.3	8.6 ± 2.6	0.15	9.3 ± 2.2	10.5 ± 2.5	0.07
	Locomotion	9.6 ± 2.1	10.2 ± 2.5	0.37	9.9 ± 2.4	11.0 ± 2.3	0.09
	Object manipulation	8.5 ± 2.1	7.8 ± 1.3	0.13	9.0 ± 2.0	8.4 ± 1.9	0.39
	GMQ	95.0 ± 12.0	92.0 ± 8.8	0.32	95.2 ± 10.4	100.2 ± 11.8	0.15
	Self-regulation	28.8 ± 18.8	28.3 ± 17.6	0.91	24.1 ± 18.2	27.3 ± 20.1	0.52
Boys	Stationary	10.1 ± 2.0	7.5 ± 2.3	0.003 *	8.6 ± 2.3	9.4 ± 2.5	0.40
	Locomotion	10.2 ± 2.2	10.1 ± 2.3	0.80	10.0 ± 1.9	10.7 ± 2.6	0.42
	Object manipulation	9.7 ± 2.3	8.1 ± 1.6	0.03 *	9.7 ± 2.1	8.2 ± 1.9	0.07
	GMQ	101.4 ± 11.3	89.2 ± 6.4	0.003 *	95.9 ± 9.6	96.6 ± 12.0	0.87
	Self-regulation	24.9 ± 18.9	27.8 ± 18.4	0.64	17.8 ± 15.8	26.9 ± 22.0	0.16
Girls	Stationary	8.9 ± 2.5	9.5 ± 2.5	0.54	10.2 ± 1.9	12.0 ± 1.7	0.03 *
	Locomotion	9.2 ± 2.1	10.2 ± 2.7	0.25	9.7 ± 3.0	11.5 ± 1.8	0.12
	Object manipulation	7.4 ± 1.2	7.5 ± 0.9	0.95	7.9 ± 1.3	8.9 ± 2.0	0.24
	GMQ	90.1 ± 10.4	94.8 ± 10.1	0.24	94.1 ± 12.0	106.7 ± 8.5	0.03 *
	Self-regulation	32.4 ± 18.5	31.2 ± 17.0	0.85	32.9 ± 18.1	27.8 ± 17.6	0.48

* Indicates statistical significance (*p* < 0.05). ^1^ Intervention and control groups were compared using independent samples *t*-tests for all outcome variables. ^2^ Note: GMQ = gross motor quotient. Standard scores of 7 to 13 are considered “average.” GMQ scores range from 85 to 115 for “average motor development.” Self-regulation scores range from 0 to 60, with higher scores indicating stronger self-regulation.

## Data Availability

The datasets generated and/or analyzed during the current study are not publicly available due participant confidentiality but are available from the corresponding author on reasonable request.

## Supplementary Materials

The following supporting information can be downloaded at https://www.mdpi.com/article/10.3390/ijerph22121861/s1, Table S1: Linear mixed-effects models for gross motor quotient and fundamental movement skill scores (coefficient (95% CI)); Table S2: Mixed-effects hurdle models for treatment effect on behavioral self-regulation scores.

## Abbreviations

The following abbreviations are used in this manuscript:

PA	Physical activity
PL	Physical literacy
SR	Self-regulation
FMS	Fundamental movement skills
ECE	Early childcare and education
PDMS-2	Peabody Development Motor Scales-Second Edition
HTKS	Head–Toes–Knees–Shoulders
GMQ	Gross motor quotient
