# Supplementary material for: Getting an Active Start: Assessing the Impact of a Physical Literacy-Based Intervention on Preschool-Aged Children’s Fundamental Movement Skills, Motor Competency and Behavioral Self-Regulation
Source: Int J Environ Res Public Health. 2025 Dec 13;22(12):1861. doi: 10.3390/ijerph22121861 (PMC12732799; doi:10.3390/ijerph22121861)
Supplement: Supplementary file 1 [file ijerph-22-01861-s001.zip › Table S1.pdf]

Table S1. Linear mixed-effects models for gross motor quotient and fundamental movement skill scores (coefficient (95% CI))

	Model 1[a]				Model 2[b]				Model 3[b]			
	S	OM	L	GMQ	S	OM	L	GMQ	S	OM	L	GMQ
**N**	109	107	107	95	103	101	99	83	103	101	99	83
**Intervention group**	-.9<br>(-2.1, .3)	-.7<br>(-1.6, .2)	.6<br>(-.5, 1.8)	-2.1<br>(-7.6, 3.4)	-.1<br>(-.8, .6)	-.1<br>(-.6, .5)	.2<br>(-.6, .9)	-.0<br>(-3.2, 3.2)	-.4<br>(-1.4, .7)	-.2<br>(-1.0, .5)	-.0<br>(-1.1, 1.1)	-.9<br>(-5.8, 4.0)
**Final timepoint**	-.2<br>(-1.1, .6)	.6<br>(-.1, 1.2)	.5<br>(-.4, 1.4)	1.7<br>(-2.4, 5.9)	-.2<br>(-1.0, .5)	.7*<br>(.1, 1.2)	.7<br>(-.1, 1.5)	3.0<br>(-.6, 6.5)	-1.4**<br>(-2.4, -.4)	.6<br>(-.3, 1.4)	.4<br>(-.9, 1.6)	-1.5<br>(-6.8, 3.7)
**Intervention*Final**	2.3***<br>(1.0, .3.6)	-.01<br>(-1.0, 1.0)	.1<br>(-1.2, 1.4)	5.6<br>(-.5, 11.6)	**2.3***<br>(1.2, 3.4)**	**-.2<br>(-1.0, .7)**	**-.3<br>(-1.5, .8)**	**3.5<br>(-1.6, 8.7)**	3.1***<br>(1.6, 4.6)	-.5<br>(-1.7, .6)	-.4<br>(-2.1, 1.2)	6.1<br>(-1.0, 13.2)
**Girls**									-.2<br>(-1.1, .8)	-.3<br>(-1.1, .5)	-.2<br>(-1.3, .9)	-1.0<br>(-5.5, 3.4)
**Intervention*Girls**									.5<br>(-1.0, 1.9)	.2<br>(-.8, 1.3)	.2<br>(-1.3, 1.7)	1.3<br>(-5.3, 7.9)
**Final*Girls**									2.4***<br>(.1, 3.7)	.2<br>(-1.0, 1.4)	.6<br>(-1.1, 2.3)	8.7*<br>(1.7, 15.7)
**Intervention*Final*Girls**									-1.6<br>(-3.7, .4)	1.2<br>(-.6, 2.9)	.5<br>(-1.8, 2.8)	-4.2<br>(-14.2, 5.9)

Note: Boldface indicates coefficients used in final interpretation (S=stationary, OM=object manipulation, L=locomotor, GMQ= gross motor quotient).

[a] Unadjusted model

[b] Adjusted for baseline child sex, age (months), BMI and dependent variable.

*p<0.05; **p<0.01; ***p<0.001
