# Supplementary material for: Getting an Active Start: Assessing the Impact of a Physical Literacy-Based Intervention on Preschool-Aged Children’s Fundamental Movement Skills, Motor Competency and Behavioral Self-Regulation
Source: Int J Environ Res Public Health. 2025 Dec 13;22(12):1861. doi: 10.3390/ijerph22121861 (PMC12732799; doi:10.3390/ijerph22121861)
Supplement: Supplementary file 1 [file ijerph-22-01861-s001.zip › Table S2.pdf]

Table S2. Mixed-effects hurdle models for treatment effect on behavioral self-regulation scores

	**Binary component Odds ratio (95% CI)**		**Positive component IRR (95% CI)**		
	**Model 1[a]**	**Model 2[b]**	**Model 3[a]**	**Model 4[b]**	**Model 5[b]**
**N**	156	134	115	103	103
**Intervention group**	.8 (.1, 5.6)	.4 (.0, 3.3)	1.0 (.7, 1.4)	1.0 (.9, 1.2)	1.1 (.8, 1.4)
**Final timepoint**	.4 (.1, 2.4)	.4 (.1, 2.8)	1.0 (.7, 1.4)	1.3 (1.0, 1.6)*	1.37 (1.0, 1.9)*
**Intervention*Final**	1.5 (.1, 16.3)	4.5 (.3, 80)	1.1 (.6, 1.8)	**1.0 (.8, 1.3)**	1.1 (.8, 1.7)
**Girls**					1.1 (.9, 1.4)
**Intervention*Girls**					1.0 (.7, 1.4)
**Final*Girls**					.9 (.6, 1.3)
**Intervention*Final*Girls**					.7 (.4, 1.2)

Note: Boldface indicates coefficients used in final interpretation.

[a] Unadjusted model

[b] Adjusted for baseline child sex, age (months), BMI and self-regulation scores.

*$p<0.05$
